# Evaluation of a patient-initiated review system in rheumatoid arthritis: an implementation trial protocol

**DOI:** 10.1186/1471-2474-13-120

**Published:** 2012-07-09

**Authors:** Priyamvada Paudyal, Mark Perry, Sue Child, Christian A Gericke

**Affiliations:** 1 PenCLAHRC, National Institute for Health Research, Peninsula Medical School, Universities of Exeter & Plymouth, B432 Portland Square, Plymouth, PL4 8AA, UK; 2 Department of Rheumatology, Plymouth Hospitals NHS Trust, Derriford, PL6 8DH, UK

## Abstract

**Background:**

Rheumatoid arthritis is a chronic inflammatory condition that affects the joints causing unpredictable episodes of pain, stiffness and disability. People with rheumatoid arthritis usually require lifelong specialist follow-up but frequently have periods when their disease can be managed through self-care or that provided by their general practitioner. Compared to the traditional clinician-driven care in rheumatoid arthritis, patient-initiated care has proven to be more beneficial in terms of reducing unnecessary medical reviews, providing greater satisfaction to patients and staffs and maintaining the patient’s physical and psychological status. We aim to evaluate the implementation of a patient-initiated review system in a routine secondary care rheumatology service in a public hospital in England, where patients get the opportunity to self-manage their disease by requesting specialist reviews at times of need instead of clinician-scheduled appointments.

**Methods/design:**

Three hundred and eighty patients attending routine review at Plymouth Hospitals NHS Trust will be randomised to either enrol immediately into a patient-initiated review system (direct access group), or to be seen regularly by a clinician at the hospital (regular clinician-initiated group). Patients (or their general practitioner) in the direct access group can arrange a review by calling a rheumatology nurse-led advice line that enables telephone delivered clinical advice, or where appropriate, an appointment with a rheumatologist within 10 working days. Patients in the regular clinician-initiated group will attend their planned appointments at regular intervals during the intervening period of 12 months. The primary outcome of interest is patient satisfaction; secondary outcomes include service use, waiting times and clinical measures. Semi-structured, in-depth interviews will be conducted with a subset of patients and staff with the aim of identifying facilitators/barriers in implementing patient-initiated clinics.

**Discussion:**

The implementation of a patient-initiated review system in routine care rheumatology will replace the fixed clinician-driven review system with a more flexible patient-driven system where patients usually self-manage their disease, but can request prompt help when required. We believe that this study will enable a comparison of the changes in local services and will be helpful in exploring the benefits/drawbacks of such implementation, thus providing lessons for implementation in other hospitals and for other chronic diseases.

## Background

Rheumatoid arthritis (RA) is a chronic disease that primarily affects the synovial joints causing unpredictable episodes of joint pain, stiffness and swelling. There are approximately 400,000 people with RA in the United Kingdom (UK), with around 15 men and 36 women developing RA per 100,000 people per year [1]. It has been estimated that the total cost associated with RA in the UK, including indirect costs and work-related disability is between £3.8 and £4.75 billion per year [1]. People with RA often have lifelong symptoms that fluctuate on a daily or longer basis, intermittent disease flares, and an unpredictable long-term outcome. Hence, these patients need to develop the skills to manage their own condition and its consequences on their lives [2].

In the UK, the majority of people with RA are managed by routine medical review initiated by the hospital rheumatologist or rheumatology specialist nurse every 3–6 months. However, this traditional system of review may lead to a mismatch between clinical need and clinical input. Patients are often seen on a date determined some time in advance, and review may occur when the patient is well rather than during periods of exacerbation [2]. A previous study into the content of routine reviews of patients with RA reported that 30% of routine appointments resulted in no investigations or other action; 35% were seen to be problem free; and 42% were thought to be unnecessary [3]. Hence, it is likely that considerable hospital out-patient time and resources are wasted on the one hand and on the other hand the systems of review may be unresponsive at the times when patients need them most.

A randomised controlled trial (RCT) conducted in Bristol, UK, compared traditional 3–6 monthly rheumatologist-initiated review with patient-initiated review alone [4-6]. This trial reported that patient-initiated reviews maintained the patient’s physical and psychological status but reduced unnecessary medical reviews by at least a third, thus making more efficient use of finite resources. The trial data also showed that patients and GPs had more confidence and satisfaction in such a system when compared to clinician- driven follow-up. Numerous studies looking at the impact of open access patient-initiated follow-up clinics in patients with medical conditions such as lung cancer [7], breast cancer [8], diabetes [9], urinary tract infections [10], gastroscopy [11] and endoscopy [12] have also reported positive effects resulting from such clinics in terms of patient care and satisfaction, cost-effectiveness and efficiency.

The National Institute for Health and Clinical Excellence (NICE) guidelines for RA recommend that people with satisfactorily controlled established RA should be able to review the frequency of their appointments at a location suitable to them and in addition, make sure they have access to additional visits for disease flares and know when and how to get rapid access to specialist care [1]. Similarly, The British Society for Rheumatology (BSR) guidelines state that patients need an individualised management plan including choices for long-term follow-up care, and self-initiated access to primary or secondary care including telephone advice [13]. These guidelines support the need for systems that promote self-management of care and treatment in patients with RA.

Given the evidence from the trial by Hewlett and colleagues [6], and the recommendations from NICE and BSR, we decided to implement a patient-initiated review system at Plymouth Hospitals NHS Trust (PHNT) in the UK. The Trust currently has a clinician-driven, overwhelmed and delayed outpatient follow up system for people with RA. We believe that the introduction of this evidence based review system will help to empower patients and provide them with the opportunity to self-manage the disease through the ability to request their own prompt specialist review when required.

## Methods

### Implementation process

Plymouth Hospitals NHS Trust serves a population of 460,000, and extrapolating from this figure it is estimated that there are about 4,500 patients with RA in the PHNT catchment area. Initially, all patients attending routine review at PHNT will be assessed for eligibility. People with RA who are 18 years or over, have had their disease for more than two years and are able to initiate telephone contact if needed will be deemed eligible. People will be excluded if they have no access to a telephone, or are thought unable themselves or through a relative or friend to initiate telephone contact via the advice line when their disease requires clinical review. All eligible patients will be educated about the patient-initiated review system prior to their enrolment into the system. However, due to the finite capacity of education sessions per week, all eligible patients will not be able to enroll into the system at once, and it will take more than twelve months to educate all the patients. Hence, we will use this timeframe as an opportunity to evaluate the new system of care against the comparator traditional clinician-driven system using a stepped-wedge study design. For this, a subgroup of patients suitable to have their care transferred to the patient-initiated review system will be randomised into two groups using computer generated numbers. The patients will either immediately enroll into the patient-initiated review system (Direct Access group (DA)), or will have regular clinician-initiated appointments (Regular Clinician initiated group (RC)) prior to transferring to the patient-initiated review system.

### Direct access group

After randomisation, people in the DA group will be provided with an initial information sheet explaining the system, and will be offered a choice of dates to attend a DA patient education session over the following weeks. The education sessions will comprise small groups of eight people with RA led by a rheumatology specialist nurse, and will focus on issues such as the operation of the DA system, what patients can expect from the system, when and how to call the advice line, when and how to ask for appointments, and any other queries regarding the system [2]. These patients will not be offered routine clinical review, and their GP will be informed about this and sent a short summary of managing common problems experienced by people with established RA. Patients or their GP can arrange prompt clinical advice and a review in clinic where required by calling a rheumatology nurse-led advice line where appointments can be accessed with a maximum delay of ten working days. The education session was piloted on fifty patients and following the feedback from the pilot session, changes were made before delivering it to the patients who are randomised. Any patients declining entry to the DA system will continue to have their care delivered through the traditional review system.

### Regular clinician initiated group

**Figure 1:**
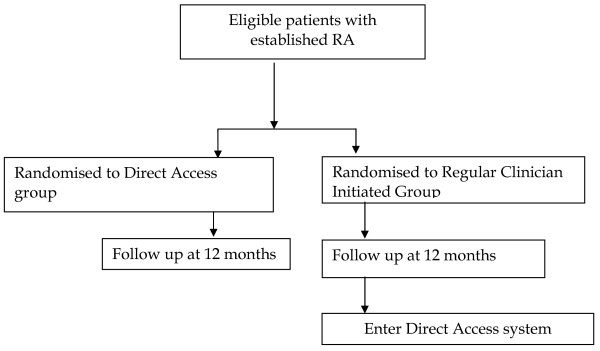
Flow diagram of the implementation trial study design.

The RC group will receive planned appointments at regular intervals over the year prior to transferring to DA at the end of the 12 months implementation process (Figure 1). Patients in both groups who do not request an appointment or who do not attend a routinely scheduled medical appointment over 12 months will be contacted for a review by the rheumatology specialist nurse.

### Implementation evaluation

The evaluation of the implementation process will be undertaken collaboratively between the PHNT Rheumatology Department and the Collaboration of Leadership and Applied Health Research and Care for the South West Peninsula (PenCLAHRC). The study will assess the following outcome measures.

### Primary outcome measure

#### Patient satisfaction

As a surrogate of service quality, patient satisfaction will be assessed at baseline and at the end of 12 months using the Short-Form Patient Satisfaction Questionnaire (PSQ-18) [14].

### Secondary outcome measures

#### Service use

Number of visits to the rheumatology consultant, rheumatology specialist nurse, physiotherapist and occupational therapist will be recorded over the period of 12 months. In a random sample of 50 patients (25 from each group), the cost of the total number of visits to hospital rheumatologists, visits to GPs and allied health professionals for problems related to arthritis and the associated travel costs will be calculated using NHS trust figures and published unit cost data [4].

#### Clinical measures

Clinical outcomes will be measured at baseline before patients enter the DA system (during the education session) and at the end of 12 months follow-up. Outcome measures will include the RA disease activity score (DAS-CRP), patient’s global opinion of disease activity (10cm scale, measured from The London Handicap Scale (LHS)), clinician’s global opinion of disease activity (10cm scale, measured from LHS), pain over the preceding 24 hours, early morning stiffness, tender joint count, swollen joint count, disability (Health Assessment Questionnaire), C reactive protein (CRP), plasma viscosity (PV), haemoglobin, rheumatoid factor (RF), rheumatoid factor titre (RFT), presence or absence of erosive disease by most recent routine X-rays, and disease duration.

#### Waiting times

As the RC group will attend the clinician-initiated outpatient clinic at regular intervals; service availability upon request will be measured for the DA group only. Waiting time will be assessed by calculating the number of days from the advice line call to the medical appointment.

Semi-structured, in-depth interviews will be carried out with a subset of patients from both groups as well as with the health professionals and administrators involved in providing the service. Interviews will particularly focus on assessing barriers and facilitators of providing a patient-initiated service, reasons for implementation success/failure, psychological benefits/drawbacks and effects on wider life impacts, and perceived or real changes in the interaction between patients and the rheumatology team delivering their medical care. All the interviews will be recorded and later transcribed. Once all the transcripts have been analysed, a matrix will be designed which will enable the emergence of major themes and create an understanding of the individual and group coding. The interviews will add depth to the analysis of quantitative data and will be supplemented through the observation of a sample of patient education sessions given by a rheumatology specialist nurse. The observation of these sessions will allow us to gather first-hand information about the social processes between the specialist rheumatology nurse and patients in an educational context. This will help inform the semi-structured interviews as the qualitative researcher will have first-hand knowledge of how patients were educated about the new appointment system and what were their early thoughts about such process change.

### Sample size and statistical analysis

The assumptions underlying the sample size calculation are derived from a published RCT [6]. In order to detect a decrease in the number of consultations from 2.5 per year in the RC group to 2 per year in the DA group at the 5% significance level with 90% power to detect this difference, we calculated that the current evaluation will require 190 subjects in each group followed up for a period of 12 months. Changes in other outcome measures between the two groups will be compared using simple tests of association such as chi-squared (*χ*^2^) test and Student’s *t*-test (depending on data properties). The qualitative data analysis technique described by Miles and Huberman [15] will be used to analyse the interview data. This means affixing codes to a set of field notes drawn from data collection and sorting through the material to identify relationships between themes. All interview recordings will be transcribed and checked for accuracy. The researcher will listen to the interview recordings and make comments on the transcript and read and re-read them to identify relevant codes. Once all transcripts have been analysed in this way a matrix will be designed which will enable the emergence of major themes and create an understanding of the individual and group coding. Computer aided software such as NVivo (a qualitative data analysis tool that helps to organise and analyse unstructured, i.e. non-numerical data) will be used to interrogate the data at different levels.

## Discussion

The implementation of the DA system in Plymouth will move the service provision from a traditional clinician-driven review system to a more flexible patient-driven system where patients can request help according to their needs. Our evaluation will provide routinely collected data enabling an assessment of the impact of implementing this type of service change in the National Health Service. In addition, the qualitative analysis will offer insights into the reasons why such a service implementation might be successful, and how both health staff and patients perceive both the benefits and drawbacks of such a change.

In the current evaluation we will be able to compare the changes in local services and inform local patients, commissioners and providers about the changes in rheumatology service provision. In the context of a previous RCT, the DA system was established as being more cost-effective, providing greater satisfaction to patients, GPs and rheumatologists, and reduced the delay in follow-up appointments compared with the traditional review system [6]. However RCTs cannot give evidence of how such a service change impacts on the delivery of healthcare in a routine setting. Our expectation is that this implementation of a patient-initiated system of care will reduce unnecessary appointments and costs, be more responsive to patients when they need rapid help, and through these processes empower patients to improve their own self-care. In the current financial climate it will be informative to see if these expectations are realised.

## Ethical approval

After advice from the National Research Ethics Service South-West (Bristol), UK, the quantitative and qualitative evaluations planned for this process do not require full, NHS ethical approval as is it an evaluation of a change in service. However, all interview respondents (both patients and professionals) will be volunteers and will all be provided with a detailed information sheet which describes the nature of the study. Written, informed consent will then be gained using a standardised consent form. Confidentiality will be emphasised at the outset and interview transcripts will be anonymised.

## Competing interests

The authors declare that they have no competing interests.

## Authors’ contribution

PP drafted the manuscript with input from all the authors. MP, CAG, SC and PP designed the study, and CAG supervised the overall study. All authors read and approved the final version of the manuscript.

## Pre-publication history

The pre-publication history for this paper can be accessed here:

http://www.biomedcentral.com/1471-2474/13/120/prepub
